# Basic Scholarship in Biosafety Is Critically Needed To Reduce Risk of Laboratory Accidents

**DOI:** 10.1128/mSphere.00010-17

**Published:** 2017-03-29

**Authors:** Ryan Ritterson, Rocco Casagrande

**Affiliations:** Gryphon Scientific, LLC, Takoma Park, Maryland, USA; University of Michigan

**Keywords:** biosafety, conduct of research, gain of function, science policy

## Abstract

Our firm conducted a risk/benefit assessment of “gain-of-function” research, as part of the deliberative process following a U.S. moratorium on the research (U.S. Department of Health and Human Services, *U.S. Government Gain-of-Function Deliberative Process and Research Funding Pause on Selected Gain-of-Function Research Involving Influenza*, *MERS*, *and SARS Viruses*, 2014). Due to significant missing but theoretically acquirable data, our biosafety assessment faced limitations, and we were forced to provide a relative, instead of absolute, measure of risk (Gryphon Scientific, LLC, *Risk and Benefit Analysis of Gain of Function Research*, 2016). Here, we argue that many of these types of missing data represent large and stunning gaps in our knowledge of biosafety and argue that these missing data, once acquired via primary research efforts, would improve biosafety risk assessments and could be incorporated into biosafety practices to reduce risk of accidents. Governments invest billions in biological research; at least a small fraction of this support is warranted to prevent biological accidents.

## Abstract (precis)

Our firm conducted a risk/benefit assessment of “gain-of-function” research, as part of the deliberative process following a U.S. moratorium on the research (U.S. Department of Health and Human Services, *U.S. Government Gain-of-Function Deliberative Process and Research Funding Pause on Selected Gain-of-Function Research Involving Influenza*, *MERS*, *and SARS Viruses*, 2014).

## PERSPECTIVE

In 2014, the White House issued a moratorium on so-called gain-of-function research involving influenza virus and severe acute respiratory syndrome (SARS) and Middle East respiratory syndrome (MERS) coronaviruses and initiated a deliberative process to be managed by the National Science Advisory Board for Biosecurity (NSABB), a process which recently concluded with the release of the White House Office of Science and Technology Policy’s (OSTP) Recommended Policy Guidance document (1, 2). In an earlier stage of the process, our firm was contracted by the National Institutes of Health to perform and deliver a risk/benefit assessment of this research to independently investigate its biosafety and biosecurity risks, as well as the scientific and public health benefits it may bring. As part of our biosafety risk assessment, we were asked to provide a measurement of the absolute risk of a biosafety incident. However, due to significant missing, but theoretically acquirable data, our work faced limitations, and we were forced to provide a relative, instead of absolute, measure of risk (3). Many of these types of missing data represent large and stunning gaps in our knowledge of biosafety. These missing data, once acquired via relatively simple primary research efforts, would not only improve biosafety risk assessments but also could be immediately incorporated into biosafety practices to reduce the risk of accidents. Governments invest billions of dollars to support biological research with the purpose of improving the human condition; clearly, at least a small fraction of this support should be used to prevent the biological accidents that imperil that basic goal.

## LACK OF HUMAN RELIABILITY DATA

To the best of our knowledge, Gryphon Scientific’s biosafety risk assessment was the first to comprehensively consider the probability and types of human error that play a role in laboratory accidents, and we identified it as the dominant component of laboratory biosafety risk. This dominance of human errors is based on multiple factors. First, most plausible pathways to laboratory-acquired infection or accidental release require a human error to precipitate. For example, spills and needlesticks begin with a person making a motor control mistake. Second, humans fail more frequently than equipment. For example, in our aerosol release scenarios, the median chance of an exhaust fan failing was estimated to be 1.5 orders of magnitude less likely than the chance of someone improperly responding to an airflow alarm should the fan fail (4). These relative probabilities align with our common sense—most laboratory scientists have likely experienced a simple human error, such as a dropped plate or tube, many times more often than we have experienced random mechanical failures. The last, but perhaps most important contribution of human error to laboratory incidents comes after an incident has already begun: human errors after an exposure, whether due to ignorance, panic, or expediency, can drastically exacerbate the consequences of that event. A laboratory worker who immediately notifies appropriate personnel and follows proper health surveillance and isolation protocols after an exposure is significantly less likely to cause secondary infections, limiting the consequences of the incident.

Despite the dominance of human error in determining risk, we could find limited data to support a quantitative estimate of human failure rates. Publicly available quantitative biosafety risk assessments done by others focused primarily on detailed measurements of equipment and mechanical failure rates, and either omitted a quantitative treatment of human error entirely (5, 6) or used a flat failure rate for all types of errors in all incidents (7). The available primary data were similarly limited, and we identified only a single source of human error measurements taken directly from biological laboratories (8). Due to these data limitations, we instead analogized the rate of human failures in laboratories to that of similar errors in other industries, primarily aerospace and nuclear power (9).

Indeed, in contrast to the life sciences, these other industries invest heavily in the study of human error. For decades, many technical industries have turned to formalized assessments of human performance and mistake rates, termed human reliability assessments (HRAs) (10, 11). These HRAs provide analytical frameworks to rigorously identify the type and frequency of the mistakes humans make, as well as the circumstances in which they are most likely to make them. Armed with the knowledge of how and when things may go wrong, risk and safety managers can work to redesign systems and update practices to best prevent common and/or serious mistakes before they occur. These investments are made even though the scale of potential consequences of an accident in the power and transportation industries is dwarfed by the consequences of a global infectious disease outbreak.

## LACK OF HISTORICAL BIOSAFETY INCIDENT DATA

Not only is scholarship lacking on how mistakes may be made, data are not collected on how mistakes have been made either. Despite a clear need for keeping these records, the United States has no standardized or comprehensive system for tracking laboratory incidents or near misses in high-containment laboratories. Astoundingly, we appear to lack even the most basic knowledge of how many high-containment laboratories are currently in operation (12). Although some partial reporting systems exist (13, 14), none of these systems are sufficiently standardized, complete, or of high-enough quality and detail to provide usable statistics about the type, magnitude, and kind of incidents that occur in biological laboratories. This lack of centralization and standardization hinders the spread of lessons learned and best practices between laboratories, which likely results in us suffering the same mistakes in multiple laboratories.

Other industries have discovered that centralized tracking of incidents can significantly reduce risk. In 1974, TWA flight 514 crashed outside Washington’s Dulles airport, killing 92 people (15). During the investigation, it was discovered that a United Airlines flight had a near miss due to the same cause just weeks earlier, and although United had alerted its pilots of the danger, the warning did not spread industry-wide. In the wake of the accident, the Federal Aviation Administration (FAA) created—and has maintained ever since—a no-fault system of reporting aviation incidents and mistakes, no matter how minor. This reporting correlates with a decrease in risk: airline industry sources show a substantial and continuing decrease in accident rates from 1976 to today (today’s rate is about a third of the rate in the early 1970s) (16, 17). Similar measures have been undertaken in the nuclear power and chemical manufacturing sectors, which greatly improve the ability to predict potential accidents and prevent them (18, 19). Although we note that these industries have had decades of additional time and experience beyond that of the relatively new life science industry to advance their state of risk assessment and prevention, we believe that the life sciences industry should strive, over time, to meet the same standards those industries have pioneered. Also, although these industries are dominated by a few giant concerns, the fact that the government and a handful of companies are responsible for the large majority of research on dangerous pathogens enables a similarly few number of influential players to effect significant change.

## BENEFITS OF GATHERING THESE DATA

The combination of prospective human reliability primary data gathering to assess what might go wrong and historical incident record keeping to assess what did go wrong provides a powerful path to reducing biosafety risk. Using these data, laboratory safety practices can be improved, lowering the risk to the researchers and to their surrounding communities. In addition, training, equipment, and safety systems can be redesigned to prevent common mistakes before they happen.

Beyond these benefits in risk reduction, gathering data on human reliability and biosafety incidents will also support the development of absolute biosafety risk assessments, which is desirable for several reasons. First, biosafety levels can begin to be defined by the maximum risk of an experiment allowable under that safety level, instead of being defined by organism phenotype, ensuring that the riskiest experiments remain tightly protected without unnecessarily burdening others. Second, absolute numbers enable comparisons across industries and activities, which contextualizes the level of biosafety risk we are assuming. By making risk less subjective, policy debates can be more focused and targeted. In our experience with the gain-of-function debate, our inability to provide absolute numbers left unresolved questions about the baseline acceptable level of risk, distracting from the policy questions at hand.

## RECOMMENDATIONS

In sum, gathering these data is achievable and would provide immense benefits. As such, we believe significantly more funding is urgently and immediately needed to support three basic thrusts: (i) development of a national incident reporting system, (ii) primary research programs focused on HRAs, equipment failures, and decontamination efficiencies, and (iii) sharing of best practices. We believe the reporting system should be structured like the FAA’s database and should include not only consequential incidents but also reports of near misses, defined as situations that required a response but where no infections or consequences resulted. Consequential incidents are often rare outcomes of a series of common mistakes or failures. By reporting near misses that may contain these same common mistakes, the mistakes can be identified and remedies can be put into place prior to the mistakes precipitating high-consequence events. The NSABB’s recommendations for overseeing gain-of-function research included—due to prompting by us and others—a recommendation to create such a database of incidents and near misses, and we strongly agree with their recommendation (20). OSTP’s guidance document in response to NSABB’s recommendations (2) does not address this data gap, and we fear momentum for the creation of the database is at risk of being lost.

Creation of such a database is not without challenges. Currently, many institutions have expressed reluctance to share information about biosafety incidents, due to fears of how that information may be shared by organizations beyond their control, for example, in the series of articles *USA Today* has published (21, 22). In addition, some institutions have hesitated to share data on incidents due to a lack of standard definitions of what qualifies as reportable, and no institution wants to be penalized as unsafe for the good deed of overreporting. Although daunting, these challenges can—and should—be overcome. One first step would be to identify the largest current disincentives to reporting and potential approaches to removing them. A second step would be building consensus for a complete definition of what should be reportable. We believe efforts to accomplish these steps should be led by the biosafety and research communities, similar to how these same communities led the efforts at Asilomar in 1975 to establish biosafety guidelines for work with recombinant DNA (23).

To address biosafety knowledge gaps, federal grant funds should be made available for primary research studies. Although the federal government does fund biosafety education and training programs, there appears to be little spending on quantitative studies of laboratory biosafety practices and accidents, despite a National Institute of Allergy and Infectious Disease (NIAID) operating budget of $4.7 billion in fiscal year 2016 (24). We note that a new annual allocation of a mere 1/10th of 1% of that number—$5 million per year—set aside for biosafety research and tracking could make significant strides to cover these gaps and greatly increase our ability to predict and prevent accidents. The funding for investigations of accidents and research into their causes by agencies whose primary mission is safety in the transportation (nearly $1 billion, via the National Transportation Safety Board [NTSB] and National Highway Traffic Safety Administration [NHTSA]) and nuclear and chemical industries (more than $1 billion via the Nuclear Regulatory Commission [NRC] and Chemical Safety Board [CSB]) demonstrates that the United States has already recognized the importance of these types of studies. In addition to studies investigating human reliability, applied, laboratory-based studies should also be conducted to gather additional data about failures due to mechanical systems or decontamination protocols. Given the recent widespread attention on failures of inactivation protocols (25, 26), learning how these protocols fail will also enhance safety and our ability to ascertain risk. Finally, although failure rates for many types of personal protective equipment (PPE) are known, many kinds of data are still lacking. Primary research in this area would be straightforward and would additionally improve biosafety risk assessments.

Although incident recordkeeping and human reliability are the two largest and most urgent areas, other areas of biosafety research have also suffered due to a lack of funding. In our experience visiting laboratories undertaking gain-of-function research, we noted some institutions maintained a strong safety culture that likely played a significant role in reducing the risk of accident in these labs. Yet, how to create this culture and the effect it has on overall safety remain understudied. In addition, we noted that some labs put into place some unique best practices, such as maintaining a separate entrance at the designated local clinic for potentially exposed laboratory personnel, limiting public exposure, yet these practices were not widely known or shared within the biosafety community. Funding for collection and dissemination of these best practices is sorely needed, would be relatively modest, and would have a nearly immediate return on investment.

Federal, state, and local governments, educational institutions, and industry participants do commit significant resources to biosafety and biorisk management, through biosafety officers, institutional biosafety committees, the *Biosafety in Microbiological and Biomedical Laboratories* manual (27), and a host of risk assessment practices, polices, and reporting requirements. These efforts play a critical role in ensuring that biological research is conducted safely. However, despite these efforts, large gaps in our knowledge on how accidents did and could occur in laboratories still exist, and this fact is surprising and inexcusable, given that an accident in a biological laboratory, while already extremely unlikely under today’s safety precautions, could lead to a global infectious disease outbreak that kills more people than all aviation and industrial accidents combined. Given the vast gaps in knowledge that exist, a significant return on investment could be expected in terms of reduced biosafety risk in the near term, making this one of the safest research investments the federal government could make. Transforming biosafety into a quantitative practice would ensure that our research enterprise can produce the cures and treatments to improve our quality of life without a single accident vitiating millions of hours of toil at the bench. It is time to make that transition a reality.
